# Agronomic biofortification of genetically biofortified wheat genotypes with zinc, selenium, iodine, and iron under field conditions

**DOI:** 10.3389/fpls.2024.1455901

**Published:** 2024-12-06

**Authors:** Hari Ram, Asif Naeem, Abdul Rashid, Charanjeet Kaur, Muhammad Y. Ashraf, Sudeep Singh Malik, Muhammad Aslam, Gurvinder S. Mavi, Yusuf Tutus, Mustafa A. Yazici, Velu Govindan, Ismail Cakmak

**Affiliations:** ^1^ Department of Plant Breeding and Genetics, Punjab Agricultural University, Ludhiana, ;India; ^2^ Nuclear Institute for Agriculture and Biology (NIAB), Faisalabad, ;Pakistan; ^3^ Pakistan Academy of Sciences, Islamabad, ;Pakistan; ^4^ Faculty of Engineering and Natural Sciences, Sabanci University Orta, Istanbul, ;Türkiye; ^5^ International Maize and Wheat Improvement Center (CIMMYT), Texcoco, ;Mexico

**Keywords:** agronomic biofortification, wheat, genotypes, micronutrients, hidden hunger

## Abstract

Inherently low concentrations of zinc (Zn), iron (Fe), iodine (I), and selenium (Se) in wheat (*Triticum aestivum* L.) grains represent a major cause of micronutrient malnutrition (hidden hunger) in human populations. Genetic biofortification represents a highly useful solution to this problem. However, genetic biofortification alone may not achieve desirable concentrations of micronutrients for human nutrition due to several soil- and plant-related factors. This study investigated the response of genetically biofortified high-Zn wheat genotypes to soil-applied Zn and foliarly applied Zn, I, and Se in India and Pakistan. The effect of soil-applied Zn (at the rate of 50 kg ha^−1^ as ZnSO_4_·7H_2_O) and foliar-applied Zn (0.5% ZnSO_4_·7H_2_O), I (0.04% KIO_3_), Se (0.001% Na_2_SeO_4_), and a foliar cocktail (F-CT: combination of the above foliar solutions) on the grain concentrations of Zn, I, Se, and Fe of high-Zn wheat genotypes was investigated in field experiments over 2 years. The predominantly grown local wheat cultivars in both countries were also included as check cultivars. Wheat grain yield was not influenced by the micronutrient treatments at all field locations, except one location in Pakistan where F-CT resulted in increased grain yield. Foliar-applied Zn, I, and Se each significantly enhanced the grain concentration of the respective micronutrients. Combined application of these micronutrients was almost equally effective in enhancing grain Zn, I, and Se, but with a slight reduction in grain yield. Foliar-applied Zn, Zn+I, and F-CT also enhanced grain Fe. In India, high-Zn genotypes exhibited a minor grain yield penalty as compared with the local cultivar, whereas in Pakistan, high-Zn wheat genotypes could not produce grain yield higher than the local cultivar. The study demonstrates that there is a synergism between genetic and agronomic biofortification in enrichment of grains with micronutrients. Foliar Zn spray to Zn-biofortified genotypes provided additional increments in grain Zn of more than 15 mg kg^−1^. Thus, combining agronomic and genetic strategies will raise grain Zn over 50 mg kg^−1^. A combination of fertilization practice with plant breeding is strongly recommended to maximize accumulation of micronutrients in food crops and to make significant progress toward resolving the hidden hunger problem in human populations.

## 1 Introduction

Deficiencies of mineral micronutrients such as zinc (Zn), iron (Fe), iodine (I), and selenium (Se) in humans are widespread globally, affecting approximately 50% of the world’s population. In many areas of the world, multi-micronutrient deficiencies are also observed, resulting in various health complications (Welch et al., 2013; Bailey et al., 2015; Harding et al., 2018; Stevens et al., 2022; FAO et al., 2023). The root cause of this type of malnutrition in the developing world is the low density of micronutrients in staple food crops, especially cereals including wheat, maize, and rice, mainly due to the low plant availability of these micronutrients in cultivated soils.

For instance, Zn deficiency in wheat is a widespread mineral nutrient disorder in India and Pakistan because the soils in these countries are alkaline and calcareous, with low organic matter and low moisture (Rashid, 2005; Cakmak, 2009; Zou et al., 2012; Ram et al., 2015). Soil Zn deficiency not only hampers crop productivity but also results in the production of low-Zn wheat, which leads to Zn malnutrition and the consequent health hazards in humans and animals (Rashid, 2005; Cakmak, 2008; Zou et al., 2012, 2019; Morton et al., 2023). In India and Pakistan, which are among the countries with the highest prevalence of hidden hunger, wheat is a major agricultural crop cultivated on over 38.0 Mha of land and represents the predominant staple cereal, with annual production of over 110 million metric tons in India and 26 million metric tons in Pakistan (Poole et al., 2021). Singh (2010) and Hussain et al. (2012) reported widespread low levels of Zn in grains, which is affecting a large segment of resource-poor families in India and Pakistan. Similarly, Kapil and Jain (2011) reported prevalence of Zn deficiency in 43.8% population of Uttar Pradesh (Northern region), Karnataka (Southern region), Orissa (Eastern region), Gujarat (Western region), and Madhya Pradesh (Central region) in India. The target populations living in rural areas consume mainly wholemeal or high extraction rate flours which, compared with “white flours” produced with a lower extraction rate, contain more Zn-rich grain components. Wheat flour currently contributes to more than 40% of Pakistan’s daily caloric intake, with per capita wheat consumption averaging around 124 kg per year, one of the highest in the world (USDA, 2014). Use of whole wheat meal in food products ensures the retention of more Zn during milling, and thus making it available to consumers because Zn is mainly concentrated in the outer aleurone layer and embryo and extremely low in the endosperm (white flour) (Cakmak et al., 2010).

Very recently, after analyzing 27 million soil tests and large human health survey studies including over one million people in India, Morton et al. (2023) reported that there is a close link between soil Zn deficiency and childhood stunting and highlighted the relevance of agronomic biofortification to minimize Zn deficiency-related health problems in India. In Pakistan, approximately 40% of the population, mostly resource-poor women and children, suffer from Zn malnutrition (NNS, 2019). Iron deficiency anemia is also a common health disorder in Pakistan; it affects 49% of mothers and 29% of children (NNS, 2019). Overall, over 1 billion of the world population are at the risk of Zn malnutrition due to inadequate dietary intake of Zn (Kumssa et al., 2015; Chen et al., 2017; Stevens et al., 2022). Intensive cropping of high-yield potential crop genotypes has aggravated the depletion of soil Zn leading to low Zn density in edible grains. Although, historically, wheat was considered less sensitive to Zn deficiency and contained high Zn concentrations in grains (Rashid and Fox, 1992; Fan et al., 2008), significant increases in wheat grain yield and grain Zn concentration have been observed with Zn fertilization in low-Zn soils in many countries of the world including India and Pakistan (Cakmak, 2008; Zou et al., 2012; Ram et al., 2015; Zou et al., 2019). So, there is a high potential for Zn enrichment of cereal grains by Zn fertilization to contribute to the alleviation of Zn deficiency problem in human populations. Iodine deficiency is another predominant public health problem around the world, persisting despite large nationwide and worldwide efforts, including the implementation of universal salt iodization and food iodine fortification programs over the past 30 years (Zimmermann and Andersson, 2012; Hatch-McChesney and Lieberman, 2022). Iodine deficiency represents a significant risk for intellectual disability and thyroid cancer (Panth et al., 2019; Smyth, 2022). A survey on worldwide I status has listed Pakistan as a country severely affected by I deficiency, with a considerable population having insufficient I intake, due to low I in soils and consequently low I in the produced food (Zia et al., 2015; Passarelli et al., 2024). Zia et al. (2015) reported very low I status in soil-plant systems in Pakistan and indicated that the origin of widespread I deficiency in human population is reduced dietary intake. Iodine-enriched cereal grains are also one of the options for curing I deficiency disorders (IDDs) in the Indian subcontinent. Several reports are available showing that soil and foliar application of I-containing fertilizers is highly effective in increasing I concentrations of the edible parts of food crops (Smoleń et al., 2016; Cakmak et al., 2017; Gonzali et al., 2017; Lyons, 2018; Budke et al., 2020). Among the treatments, foliar spray of I-containing fertilizers such as KIO_3_ has been shown to be very effective in improving the grain I concentration of cereal crops (Zou et al., 2019; Prom-u-thai et al., 2020; Cakmak et al., 2020)

Selenium (Se), being a constituent of selenoproteins, is another essential mineral micronutrient for humans. Selenium has a number of critical physiological functions in mammalian systems including the healthy functioning of the immune system, anti-inflammatory effects, preventive effects on various types of cancers, and antioxidative impacts under stressful conditions (Rayman, 2000; 2012; Lyons et al., 2005; Sun et al., 2023). It is estimated that approximately one billion people worldwide are affected from Se deficiency (Lyons et al., 2005; Jones et al., 2017). Very low levels of Se in staple food crops and consequently low dietary intake of Se are known to be the major reasons behind the widespread Se deficiency in human populations (Broadley et al., 2006; Rayman, 2008; Hurst et al., 2013). This justifies greater attention being given to the enrichment of food crops with Se, for example, by agronomic biofortification. There are well-documented examples demonstrating significant effects of soil or foliar applied Se fertilizers in enriching food crops with Se (Broadley et al., 2006; Schiavon et al., 2020; Prom-u-thai et al., 2020; Cakmak et al., 2020; Daud et al., 2024).

Deficiencies of all these micronutrients in humans and animals is exacerbated by the high-yielding Green Revolution cereal varieties, which often produce less micronutrient-dense grains than previously grown cultivars (Fan et al., 2008; Dobermann et al., 2022). Therefore, biofortification of staple crops, like wheat, to achieve higher micronutrient concentrations in grains represents an important humanitarian challenge. Development of wheat varieties with higher grain micronutrient density through plant breeding approaches is widely considered as a promising and cost-effective approach for eliminating micronutrient malnutrition worldwide (Welch and Graham, 2004; Bouis and Saltzman, 2017). Significant progress has been made in the targeted development and deployment of biofortified wheat with enhanced Zn concentrations, reaching more than two million households with biofortified wheat varieties in South Asia (Govindan et al., 2019). This effort has involved transferring high-Zn genes from selected landraces, spelt wheat and emmer wheat-derived synthetic wheat, to increase grain Zn levels of high-yielding wheat varieties by 30%–40% while preserving their agronomic competitiveness across different geographies (Guzmán et al., 2014). The identification of new genetic sources of high Zn was critical due to the limited variation and low absolute content of this micronutrient in the grain of hexaploid elite wheat lines. This targeted breeding for improved Zn content led to the release of more than 20 Zn-biofortified wheat varieties in target countries. All these varieties exhibit yields, which are at least as high as the conventional varieties released in the same regions, and an average increase of 8 ppm–10 ppm (25%–40%) in Zn level in the grain (Govindan et al., 2022). CIMMYT-derived Zn-biofortified wheat varieties are already in farmers’ fields, occupying nearly 2 million hectares and have been the subject of nutrition studies that demonstrate a reduction in child morbidity associated with their consumption (Sazawal et al., 2018).

Biofortified wheat has several potential advantages as a delivery vehicle for essential micronutrients in developing countries. In India and Pakistan, CIMMYT-derived wheat varieties, which are rapidly and widely adopted in both the countries, are excellent delivery vehicles for high Zn wheat, and the self-pollinated, true-breeding nature of the species means that the trait is likely to be stable for several generations of on-farm seed production. A few biofortified wheat genotypes developed at the International Maize and Wheat Improvement Center (CIMMYT) under the HarvestPlus project with high Zn concentrations were commercialized in India and Pakistan. Those biofortified wheat cultivars including PBW-1-Zn in India, and Zincol-2016, Akbar-2019, and Nawab-2021 in Pakistan have been recommended for general cultivation. These genotypes were developed from crosses between high-yielding parents and genetic resources containing high Zn (Govindan et al., 2012).

It is, however, important to highlight that genetic biofortification alone, as in the case of wheat, may not achieve desirable concentrations of micronutrients for human nutrition if the cultivated soils have several physical and chemical limitations reducing phytoavailability and root uptake of micronutrients such as low soil moisture, low organic matter, extreme soil pH values, and reduced root colonization with arbuscular mycorrhizal fungi as shown for Zn (Cakmak, 2008; Yazici et al., 2021). Accordingly, Zn concentrations in grains of the Zn-biofortified wheat varieties are still not sufficiently high to meet required human dietary intake for Zn. Limited genetic variation within the germplasm for the targeted micronutrients and decreases in yield potential associated with genetic improvements in grain concentrations represent with limitations (Cakmak, 2008; Hussain et al., 2012; Rashid et al., 2023; Ishfaq et al., 2023). More importantly, to our knowledge no example of multimicronutrient-biofortified variety of any crop exists. These observations also indicate that there is a high need for further enhancement of grain Zn density and to biofortify the Zn-biofortified wheat cultivars with other micronutrients, including I and Se. Considering the successful examples described above, agronomic biofortification represents an excellent strategy for further enhancements of Zn as well as other micronutrients in grains of genetically biofortified genotypes. This study investigated the response of HarvestPlus-biofortified high-Zn wheat genotypes to soil applied Zn and foliarly applied Zn, I, and Se in India and Pakistan under field conditions.

## 2 Materials and methods

### 2.1 Field locations and soils of the experiment

Field experiments were conducted at three locations in India, viz., Ludhiana (30.9010° N, 75.8573° E), Gurdaspur (32.0414° N, 75.4031° E), and Bathinda (30.2110° N, 74.9455° E), during 2015–2016 and 2016–2017, in Pakistan at Faisalabad (31.4504° N, 73.1350° E) during 2015–2016 and 2016–2017, and in Gujranwala (32.1877° N, 74.1945° E) and Sheikhupura (31.7167° N, 73.9850° E) during 2016–2017. Some salient characteristics of the experimental field soils are presented in **Table 1**. Soils of all field sites in both countries were low in DTPA-extractable Zn (0.42 mg kg^−1^–0.59 mg kg^−1^), except for Gujranwala in Pakistan in 2016–2017 where the soil contained 0.71 mg Zn kg^−1^. Fertilizer nutrients applied (per hectare) to each treatment were 150 kg N, 17.5 kg P, and 25 kg K in India and 120 kg N, 34.8 kg P, and 41.5 kg K in Pakistan. In India, the crop was sown during 7–28 November and harvested during 17–29 April in both years. In Pakistan, the sowing and harvesting dates were during 21–28 November and 20 April–04 May, respectively.

**Table 1:** Salient properties of the experimental field soils.

Country	Location	DTPA-extractable Zn (mg kg^-1^)	EC_1:1_ (dS m^−1^)	pH_1:1_ (water)	Organic matter (%)
India	Ludhiana	0.51	0.14	7.7	0.31
	Gurdaspur	0.59	0.23	7.2	0.40
	Bathinda	0.42	0.45	8.2	0.29
Pakistan	Faisalabad	0.42	0.84	7.8	1.08
	Gujranwala	0.71	0.65	7.9	0.43
	Sheikhupura	0.55	0.38	8.2	0.46

### 2.2 Experimental treatments

The field experiment evaluated the following five micronutrient treatments: (i) local control (LC, i.e., soil application of recommended rates of N, P, and K only); (ii) LC + soil Zn (S-Zn, i.e., 50 kg ZnSO_4_·7H_2_O ha^−1^); (iii) LC + foliar Zn (F-Zn, i.e., 0.5% solution of ZnSO_4_·7H_2_O); (iv) LC + foliar I (F-I, i.e., 0.04% solution of KIO_3_); and (v) foliar cocktail (F-CT, i.e., a combined solution of 0.5% ZnSO_4_·7H_2_O + 0.04% KIO_3_ + 0.001% Na_2_SeO_4_). All spray solutions contained 0.05% detergent powder (*Surf*) as surfactant. Three wheat genotypes were used in the experiments in each country: CIMMYT-derived PBW-1-Zn, HPBW-10, and locally well-adapted cv. HD-2967 in India and CIMMYT-derived Zincol-2016, NR-488, and locally well-adapted cv. Faisalabad-2008 in Pakistan. The parentages of the genotypes are given in **Table 2**. The experiment was conducted in split plot design with four replicates, by allocating micronutrient treatments in main plots and wheat genotypes in subplots. The size of each experimental subplot was 10 m^2^. Wheat was sown using a specialized hand sowing two-tined drill fitted with a seed metering device, adjusted to a seed sowing rate of 100 kg ha^−1^. Soil Zn was broadcast applied just prior to crop sowing, and all foliar treatments were applied twice, i.e., at ear initiation and early grain milk stage. Foliar sprays, at the rate of 500 L per hectare, were applied very late in the afternoon or under cloudy day to minimize rapid evaporation of the sprayed solution as described by Zou et al. (2019).

**Table 2:** Parentage of the genotypes/varieties used in the study.

Variety	Parentage
PBW 1 Zn	T. dicoccumC19309/Ae. squarrosa(409)/3/MILAN/S87230//BAV92/4/2*MILAN/S87230//BAV 92
HPBW 10	INQALAB91*2/TUKURU//WHEAR/6/BAV92//IRENA/KAUZ/3/HUITES/4/T.SPELTAPI348764/5/BAV92//IRENI/KAUZ/3/HUITES
HD 2967	ALONDRA/CUCKOO//URES81/HD-2160-M/HD-2278
NR-488	T.DICOCCON CI9309/AE.SQUARROSA (409)/3/MILAN/S87230//BAV92/4/2*MILAN/S87230//BAV92
Zincol-2016	OASIS/SKAUZ//4*BCN/3/2*PASTOR/4/T.SPELTAPI348449/5/BAV92/3/OASIS/SKAUZ//4*BCN/4/PASTOR/6/WBLL1*2/CHAPIO
Faisalabad-2008	PBW65/2*Pastor

### 2.3 Grain yield

At maturity, the crop was harvested from each plot, avoiding border rows. After allowing a few days to dry in the sun, the crop was threshed and grain yield was recorded. Grain yield was adjusted to account for 13% moisture content. Wheat grains were sampled from each plot for analysis of Zn, Fe, I, and Se.

### 2.4 Grain analysis for micronutrients

The grain samples collected were first washed with tap water and then with deionized water to remove the adhering soil dusts and other possible contaminants. The washed samples were dried at approximately 40°C in a forced-draft oven to a constant weight. For analysis of Zn, Fe, and Se, dried grains were digested with nitric acid (HNO_3_, Suprapure 65%) in a microwave-accelerated reaction system (MARS 6, CEM Corp., USA) and diluted to 100 mL. For measuring the I concentration, the grains (250 ± 10 mg) were extracted in tetramethyl-ammonium hydroxide (TMAH; 20 mL 1.25%) at 90°C using a closed-vessel microwave reaction system (MARS 6, CEM Corp., USA), as described by Cakmak et al. (2017). The cooled extracts were centrifuged for 20 min at 5,000 rpm, diluted to 1:1 by volume with double-deionized water (4 μS cm^−1^ at 25°C), and filtered in capped plastic vials. The measurement of Zn and Fe concentrations in the acid digests was made by inductively coupled plasma optical emission spectroscopy (ICP-OES; Vista-PRO Axial; Varian Pty Ltd; Mulgrave, Australia) and of I and Se concentrations by inductively coupled plasma mass spectrometry (ICP-MS; 7700 Series, Agilent Technologies, USA).

### 2.5 Statistical analysis

The data were statistically analyzed following two-way analysis of variance (ANOVA), with variety and soil/foliar micronutrient treatment as the two factors, and means were compared by protected least significance difference (protected LSD) test at a confidence level of 95%. The statistical analyses were performed using computer-based SAS software (SAS 8.0, USA).

## 3 Results

### 3.1 Grain yield

Wheat grain yield was not affected by any of the micronutrient treatments in both years at all field locations, except at Faisalabad in Pakistan during 2015–2016 (**Table 3**). During 2015–2016 in Faisalabad, Pakistan, foliar cocktail (F-CT) application resulted in significantly higher grain yield than the local control (LC). Averaged across soil/foliar treatments, except at Gurdaspur in 2015–2016, the grain yield of wheat genotypes in India did not differ from each other at all field locations, whereas in Pakistan, grain yield differed significantly among the wheat genotypes during both the years. At Gurdaspur (India) in 2015–2016, no grain yield of the biofortified high-Zn genotypes could surpass the yield of Indian check variety cv. HD 2967. In Pakistan during 2015–2016, averaged across all micronutrient treatments, maximum grain yield was recorded for the HarvestPlus wheat line NR-488 (3.52 Mg ha^−1^), which was significantly higher than Zincol-2016 and Faisalabad-2008 at Faisalabad. During 2016–2017, the check variety cv. Faisalabad-2008 in Pakistan had the highest grain yield across all experimental sites, followed by HarvestPlus-genotype Zincol-2016 except at the Faisalabad location in 2015–2016.

**Table 3:** Effect of micronutrient fertilizer treatments on grain yield of different wheat genotypes grown at various locations in India and Pakistan.

Treatment/genotype	Grain yield (Mg ha^−1^) India					
	Ludhiana	Gurdaspur	Bathinda	Ludhiana	Gurdaspur	Bathinda
	2015-16			2016-17		
Soil/foliar						
LC	5.33	5.24	4.47	5.79	4.95	4.41
LC + S-Zn	5.19	5.31	4.44	5.96	5.14	4.82
LC + F-Zn	5.23	5.03	4.24	5.97	5.34	4.96
LC + F-Zn+I	4.88	4.98	4.07	6.04	4.94	4.98
LC + F-CT	4.95	4.58	4.00	6.07	4.79	4.42
LSD_T_ (p=0.05)	n.s.	n.s.	n.s.	n.s.	n.s.	n.s.
Genotype						
PBW 1 Zn	5.00	4.97b	4.33	5.99	4.99	4.64
HPBW 10	5.16	4.95b	4.09	5.93	5.03	4.71
HD 2967	5.18	5.16a	4.31	5.97	5.09	4.80
LSD_G_ (p=0.05)	n.s.	0.15	n.s.	n.s.	n.s.	n.s.
LSD_G×T_ (p=0.05)	n.s.	n.s.	n.s.	n.s.	n.s.	n.s.

	Pakistan			
	Faisalabad		Gujranwala	Sheikhupura
	2015–2016	2016–2017	2016–2017	2016–2017
Soil/foliar				
LC	2.79 b	6.01	5.39	4.67
LC + S-Zn	2.89 b	6.00	5.39	4.47
LC + F-Zn	2.94 b	5.70	5.22	4.58
LC + F-Zn+I	–	5.93	5.05	4.23
LC + F-CT	3.22 a	6.03	5.26	4.54
LSD_T_ (p=0.05)	0.16	n.s.	n.s.	n.s.
Genotype				
Zincol-2016	2.73 b	5.47 c	4.72 b	4.54 b
NR-488	3.52 a	5.90 b	4.66 b	3.93 c
FSD-2008	2.62 b	6.43 a	6.41 a	5.02 a
LSD_G_ (p=0.05)	0.19	0.33	0.37	0.31
LSD_G×T_ (p=0.05)	n.s.	n.s.	n.s.	n.s.

Different letters across a column indicate significant differences according to LSD test (p<0.05). Where the letters are missing, ANOVA revealed the absence of an overall effect. n.s. stands for not significant.

LC, local treatment; S-Zn, soil Zn application; F-Zn, foliar Zn application; F-Zn+I, foliar Zn and I (iodine) application; and F-CT, combined foliar treatment of Zn+Se+I.

Based on the pooled means across years and locations, significant reductions of 3.9% and 6.9% in grain yield were recorded in India with foliar Zn+I (F-Zn+I) and F-CT treatments, respectively (**Table 4**). Soil application of Zn (S-Zn) resulted in the highest grain yield, which was 2.5% higher than with local control treatment. In contrast, based on the pooled means of 2016–2017 across locations in Pakistan, the grain yield was non-significantly affected by F-Zn+I and F-CT treatments, with decreases of only 2.1% and 1.1%, respectively, as compared with the local control (LC). Based on the pooled analysis, the local check variety of India, i.e., HD 2967, produced the highest grain yield. The grain yield of HarvestPlus-genotype PBW-1-Zn was similar to HD 2967, but HPBW 10 had the lowest grain yield. In Pakistan, Zincol-2016 and NR-488 produced 17.5% and 18.4% less grain yield than the local check variety Faisalabad-2008. Based on the pooled analysis across all field locations in India and Pakistan in 2016–2017, interaction between genotypes and treatments was non-significant. A slight reduction in grain yield, i.e., 3.1% with F-CT and 4.8% with F-Zn+I, was recorded across the genotypes and countries.

**Table 4:** Pooled means of grain yield across field locations and years in India and Pakistan in 2016–2017.

Genotype	Grain yield (Mg ha^−1^)					
	LC	S-Zn	F-Zn	F-Zn+I	F-CT	Mean
	India					
PBW 1 Zn	5.15	5.11	5.11	4.86	4.76	5.00A
HPBW 10	4.99	5.17	5.03	4.83	4.93	4.99B
HD 2967	5.16	5.22	5.25	4.97	4.86	5.09A
Mean	5.10A	5.17A	5.13A	4.89B	4.85B	
	Pakistan					
Zincol-2016	5.02	4.91	4.95	4.77	4.89	4.91 B
NR-488	4.90	4.87	4.83	4.64	4.91	4.83 B
FSD-2008	6.11	6.09	5.72	5.81	6.02	5.95 A
Mean	5.35	5.29	5.17	5.07	5.28	
Country Mean	5.23	5.23	5.15	4.98	5.07	
LSD (p=0.05)		India		Pakistan		
Treatment		0.18		n.s.		
Genotypes		0.09		0.20		
Interaction (T×G)		n.s.		n.s.		

Different letters across mean values of a country indicate significant differences according to LSD test (p<0.05). Where the letters are missing, ANOVA revealed the absence of an overall effect. n.s. stands for not significant.

LC, local treatment; S-Zn, soil Zn application; F-Zn, foliar Zn application; F-Zn+I, foliar Zn and I (iodine) application; and F-CT, combined foliar treatment of Zn+Se+I

### 3.2 Grain zinc concentration

The zinc concentration in wheat grains differed significantly among the micronutrient treatments at all field locations during both years in India and Pakistan, except at Bathinda in India during 2016–2017 where it varied non-significantly (**Table 5**). In India during both years, the grain Zn concentration was consistently highest with the application of F-CT at all field locations; however, all the foliar Zn treatments (F-Zn, F-Zn+I, and F-CT) were statistically at par with each other at Ludhiana. At Gurdaspur during 2015–2016, the grain Zn concentration with F-CT treatment was more than double of the recorded value with local control treatment. In India, at Gurdaspur and Bathinda during 2015–2016 and at Bathinda during 2016–2017, soil application of Zn did not improve the grain Zn concentration as compared with control. In Pakistan during 2015–2016, F-Zn treatment resulted in the highest grain Zn concentration at Faisalabad (51.7 mg kg^−1^), which was significantly higher than the Zn concentrations obtained with local control and S-Zn treatments. Foliar application of cocktail (F-CT) also resulted in higher grain Zn concentration than local control and S-Zn treatments. At the same field site in Pakistan, during 2016–2017, application of F-Zn and F-Zn+I resulted in significantly higher grain Zn concentrations than the local control as well as S-Zn and F-CT treatments. Except for Faisalabad during 2015–2016, soil Zn application did not improve grain Zn concentration as compared with local control.

**Table 5:** Effect of micronutrient treatments on grain Zn concentrations of different wheat genotypes grown at various locations in India and Pakistan.

Treatment/genotype	Zn concentration (mg kg^−1^) India					
	Ludhiana	Gurdaspur	Bathinda	Ludhiana	Gurdaspur	Bathinda
	2015–2016			2016–2017		
Soil/foliar						
LC	34.3c	25.1d	31.2d	30.9c	26.7e	33.7
LC + S-Zn	38.4b	28.4d	31.9d	39.5b	27.9d	33.6
LC + F-Zn	50.3a	45.6c	42.8c	51.1a	38.6c	41.7
LC + F-Zn+I	51.0a	49.8b	45.9b	50.2a	40.8b	47.3
LC + F-CT	52.9a	57.9a	50.6a	53.1a	42.3a	38.8
LSD_T_ (p=0.05)	2.8	3.4	2.8	3.7	0.9	n.s.
Genotype						
PBW 1 Zn	47.6a	44.2a	43.3a	47.6a	41.3a	38.9
HPBW 10	48.9a	41.9a	37.8b	46.1a	31.2c	39.9
HD 2967	39.6b	37.9b	40.2a	41.2b	33.3b	38.3
LSD_T_ (p=0.05)	2.3	3.2	2.8	2.3	1.3	n.s.
LSD_T×G_ (p=0.05)	n.s.	n.s.	n.s.	n.s.	n.s.	n.s.

	Pakistan			
	Faisalabad		Gujranwala	Sheikhupura
	2015–2016	2016–2017	2016–2017	2016–2017
Soil/foliar				
LC	36.8 c	32.8 c	27.3 c	32.0 c
LC + S-Zn	39.3b	34.2 c	29.2c	33.3 c
LC + F-Zn	51.7 a	51.9 a	52.1a	52.7 a
LC + F-Zn+I	–	53.8 a	53.3 a	53.5 a
LC + F-CT	50.4 a	46.8 b	43.9 b	46.5 b
LSD_T_ (p=0.05)	2.34	2.63	3.29	4.00
Genotype				
Zincol-2016	45.3 b	42.3 b	38.1 c	48.6 a
NR-488	48.3 a	47.7a	44.7 a	44.2b
FSD-2008	40.0 c	41.7b	40.9b	38.1c
LSD_G_ (p=0.05)	2.03	2.04	2.55	3.10
LSD_T×G_ (p=0.05)	n.s.	n.s.	n.s.	n.s.

Different letters across a column indicate significant differences according to LSD test (p<0.05). Where the letters are missing, ANOVA revealed the absence of an overall effect. n.s. stands for not significant.

LC, local treatment; S-Zn, soil Zn application; F-Zn, foliar Zn application; F-Zn+I, foliar Zn and I (iodine) application; and F-CT, combined foliar treatment of Zn+Se+I.

In India, the studied wheat genotypes differed significantly with respect to grain Zn concentration at all locations, except at Bathinda in 2016–2017. The maximum grain Zn concentration was generally recorded in the HarvestPlus-biofortified genotype PBW-1-Zn, which was significantly higher than local check variety HD 2967 at all the locations, except at Bathinda where the Zn concentration was similar in both the varieties. Among wheat genotypes studied in Pakistan, the highest grain Zn concentration was found in the HarvestPlus-biofortified genotype NR-488 at Faisalabad during 2015–16 and at Faisalabad and Gujranwala during 2016–2017, and it was significantly higher than Zincol-2016 and the local check Faisalabad-2008. In Sheikhupura during 2016–2017, Zincol-16 had the highest grain Zn concentration among the genotypes tested.

Based on the pooled analysis across locations and years in India, F-CT resulted in the highest grain Zn concentration (49.3 mg kg^−1^), which was statistically at par with F-Zn+I (**Table 6**). Compared with Zn concentrations recorded with local control treatment, F-Zn resulted in 48.5% higher Zn concentration, F-Zn+I in 56.8% higher concentration, and F-CT in 62.7% higher Zn concentration. In Pakistan, on average, the highest increase in grain Zn concentration (43%) was recorded with F-Zn+I and the lowest increase was observed with soil Zn application. The grain Zn concentration found with F-Zn+I was similar to that with F-Zn, but significantly higher than the rest of the treatments.

**Table 6:** Pooled means of grain zinc concentration in wheat genotypes across locations in India and Pakistan in 2016–2017.

Genotype	LC	S-Zn	F-Zn	F-Zn+I	F-CT	Mean
	Zn concentration (mg kg^-1^) India					
PBW 1 Zn	32.5	35.4	48.4	50.6	52.3	43.8A
HPBW 10	29.8	33.4	44.3	48.2	49.2	41.0B
HD 2967	28.7	31.0	42.3	43.8	46.3	38.4C
Mean	30.3C	33.3C	45.0B	47.5AB	49.3A	
	Pakistan					
Zincol-2016	33.0 e	34.4 e	52.4 bc	50.7 c	44.4 d	42.9 B
NR-488	31.4 ef	33.3 e	54.5 b	57.5 a	51.0 c	45.5 A
FSD-2008	27.6 g	29.6 fg	49.7 c	52.0 bc	41.8 d	40.1 C
Mean	30.7 C	32.4 C	52.2 A	53.4 A	45.7 B	
Country mean	30.5	32.9	48.6	50.4	47.5	
LSD (p=0.05)		India		Pakistan		
Treatment		4.0		1.3		
Genotypes		2.2		1.7		
Interaction (T×G)		n.s		3.0		

Different letters across mean values of a country indicate significant differences according to LSD test (p<0.05). Where the letters are missing, ANOVA revealed the absence of an overall effect. n.s. stands for not significant.

Among all wheat genotypes in India, the genotype PBW-1-Zn exhibited the highest grain Zn concentration, which was statistically higher than the grain Zn concentration of HPBW 10 and HD 2967 genotypes. PBW-1-Zn had 14.1% higher Zn and HPBW 10 had 7.5% higher grain Zn concentrations, compared with the grain Zn concentration of the local check cultivar (i.e., HD 2967) (p=0.05). Similarly, HarvestPlus-developed high-Zn genotypes NR-488 and Zincol-2016 had significantly higher grain Zn than the local check cv. Faisalabad-2008 in Pakistan. The effect of the Zn treatments on grain Zn concentration was cultivar-specific in Pakistan. In the HarvestPlus-biofortified genotype NR-488, the enhancement in grain Zn concentration was maximum with F-Zn+I application and was significantly higher than that recorded for the HarvestPlus-biofortified genotype Zincol-2016 and local check cv. Faisalabad-2008 under the same treatment. Enhancement in grain Zn concentration with foliar Zn, as F-Zn or F-Zn+I, was higher in the HarvestPlus-biofortified genotypes than the local check variety. The measured grain Zn concentrations were close to each other for the F-Zn treatment (i.e., 48.6 mg kg^−1^), F-Zn+I (i.e., 50.4 mg kg^−1^), and F-CT (i.e., 47.5 mg kg^−1^) in the mean data from both countries.

### 3.3 Grain iron concentration

Although Fe was not applied, there was a significant variation in grain Fe concentrations among the experimental treatments at all field locations in India, except at Bathinda during 2016–2017 (**Table 7**). The highest grain Fe concentration was recorded with the F-CT treatment at all field locations during 2015–2016 and at Ludhiana during 2016–2017. At Gurdaspur, the grain Fe concentrations with the three foliar treatments (F-CT, F-Zn, and F-Zn+I) were statistically similar, and all were significantly higher than the control and S-Zn treatments. The grain Fe concentration also differed significantly among wheat genotypes across field locations and years, except at Gurdaspur (2015–2016) and Bathinda (2016–2017) locations in India. During 2015–2016, at Ludhiana the genotype PBW-1-Zn had significantly higher grain Fe concentration compared with rest of the genotypes. However, at Bathinda (India), the grain Fe concentration of genotype PBW-1-Zn was significantly higher compared with genotype HPBW 10, but was similar to HD 2967. During 2016–2017 at Ludhiana (India), the grain Fe concentrations in PBW-1-Zn (30.0 mg kg^−1^) and HPBW 10 were statistically similar, and higher than that of HD 2967. At Gurdaspur in India during 2016–2017, the grain Fe concentration in PBW-1-Zn (33.8 mg kg^−1^) was higher than the other two genotypes.

**Table 7:** Effect of micronutrient treatments on grain Fe concentrations of various wheat genotypes grown at different locations of India and Pakistan.

Treatment/genotype	Fe concentration (mg kg^−1^) India					
	2015–2016			2016–2017		
	Ludhiana	Gurdaspur	Bathinda	Ludhiana	Gurdaspur	Bathinda
Soil/foliar						
LC	26.5b	27.8c	23.9b	27.9b	29.3b	32.2
LC +S-Zn	26.3b	29.6c	22.3b	28.4b	27.9b	32.0
LC +F-Zn	25.7b	34.9b	23.2b	29.0ab	32.1a	32.9
LC +F-Zn+I	26.8b	34.4b	23.1b	29.8ab	33.4a	33.7
LC +F-CT	32.0a	39.1a	25.4a	30.3a	31.7ab	34.2
LSD_T_ (p=0.05)	1.9	2.4	1.7	1.4	1.5	n.s.
Genotype						
PBW 1 Zn	30.4a	33.9	24.9a	30.0a	33.2a	33.2
HPBW 10	26.8b	33.5	22.0b	29.8a	28.3c	33.2
HD 2967	25.2c	32.1	23.8a	27.5b	31.1b	32.6
LSD_G_ (p=0.05)	2.0	n.s.	1.3	1.2	1.7	n.s.
LSD_T×G_ (p=0.05)	n.s	n.s	n.s	n.s	n.s	n.s

	Pakistan			
	Faisalabad		Gujranwala	Sheikhupura
Soil/foliar	2015–2016	2016–2017	2016–2017	2016–2017
LC	39.4 c	36.5 b	37.1 c	33.8 b
LC +S-Zn	42.1 c	37.0 b	38.2 bc	33.9 b
LC +F-Zn	47.9 b	37.9 b	38.9 abc	35.7 ab
LC +F-Zn+I	–	38.2 b	39.7 ab	35.1 ab
LC +F-CT	51.7 a	40.6 a	41.0 a	36.9 a
LSD (p=0.05)	3.3	2.0	2.3	2.5
Genotype				
Zincol-2016	45.9	38.3	37.0 c	36.5 a
NR-488	44.1	37.7	39.9 b	35.5 a
FSD-2008	45.9	38.3	41.0 a	33.1 b
LSD_G_ (p=0.05)	n.s.	n.s.	1.8	1.9
LSD_T×G_ (p=0.05)	n.s.	3.4	n.s.	n.s.

Different letters across a column indicate significant differences according to LSD test (p<0.05). Where the letters are missing, ANOVA revealed the absence of an overall effect. n.s. stands for not significant.

LC, local treatment; S-Zn, soil Zn application; F-Zn, foliar Zn application; F-Zn+I, foliar Zn and I (iodine) application; and F-CT, combined foliar treatment of Zn+Se+I

In Pakistan, F-CT consistently resulted in the highest grain Fe concentration among the treatments; however, the concentration values differed non-significantly with the other foliar treatments (F-Zn and F-Zn+I) at Gujranwala and Sheikhupura during 2016–2017 (**Table 7**). At Faisalabad during both the years, the grain Fe concentration differed non-significantly among the tested genotypes. During 2016–2017, the grain Fe concentrations of HarvestPlus high-Zn wheat genotypes (Zincol-2016 and NR-488) were statistically lower than standard check Faisalabad-2008 at Gujranwala, whereas a reverse was found true at Sheikhupura.

Pooled means of grain Fe concentrations revealed that F-CT and F-Zn+I treatments in India and F-CT, F-Zn+I, and F-Zn treatments in Pakistan significantly enhanced the grain Fe concentration (**Table 8**). In Pakistan, F-CT treatment resulted in the highest (19%) increase in grain Fe concentration than the local control treatment. Pooled analysis of grain Fe concentrations also revealed that all genotypes in India contained similar grain Fe concentrations. In Pakistan, genotype NR-488 had the highest grain Fe concentration with F-CT than other soil/foliar treatments. F-CT recorded 3.9 mg kg^−1^ higher grain Fe than the local control treatment.

**Table 8:** Pooled means of grain Fe concentration in wheat grains across locations during both years in India and Pakistan in 2016–2017.

Genotype	LC	S-Zn	F-Zn	F-Zn+I	F-CT	Mean
	Fe concentration (mg kg^−1^) India					
PBW 1 Zn	29.8	29.3	30.8	30.7	34.1	30.9
HPBW 10	26.8	26.9	28.0	30.5	32.5	28.9
HD 2967	27.3	27.1	30.0	29.4	29.8	28.7
Mean	28.0B	27.8B	29.6B	30.2A	32.1A	
	Pakistan					
Zincol-2016	36.2 cd	37.7 bc	37.5 bc	36.9 BCD	37.8 bc	37.2
NR-488	35.1 d	35.0 d	37.0 bcd	38.1 bc	41.9 a	37.4
FSD-2008	36.1 cd	36.1 cd	38.2 bc	38.3 b	38.6 b	37.5
Mean	35.8 C	36.3 C	37.6 B	37.8 B	39.4 A	
Country mean	31.9	32.1	33.6	34.0	35.8	
LSD (p=0.05)		India		Pakistan		
Treatment		1.9		1.2		
Genotypes		n.s		n.s		
Interaction (T×G)		n.s		2.1		

Different letters within a column and across mean values of a country indicate significant differences according to LSD test (p<0.05). Where the letters are missing, ANOVA revealed the absence of an overall effect. n.s. stands for not significant.

### 3.4 Grain iodine

The wheat grain I concentration substantially increased with all micronutrient treatments containing I, in both the countries during both the years (**Table 9**). As expected, the lowest grain I concentrations were found with the control treatment (LC). The F-CT and/or F-Zn+I treatments resulted in significantly higher grain I concentration than the local control treatment at all field locations during both the years. During 2016–2017, at Gurdaspur and Bathinda in India and at Sheikhupura in Pakistan, grain I concentrations with F-CT and F-Zn+I were statistically at par with each other.

**Table 9:** Effect of micronutrient treatments on grain I concentration of various wheat genotypes grown at different locations of India and Pakistan.

Treatment/genotype	I Concentration (µg kg^−1^) India					
	2015–2016			2016–2017		
	Ludhiana	Gurdaspur	Bathinda	Ludhiana	Gurdaspur	Bathinda
Soil/foliar						
LC	19c	6c	26c	10c	6b	9b
LC +F-Zn+I	455b	271b	211b	329b	220a	250a
LC +F-CT	727a	481a	310a	491a	207a	255a
LSD_T_ (p=0.05)	89	98	100	66	102	89
Genotype						
PBW 1 Zn	360	263a	193	256b	141ab	175a
HPBW 10	408	163b	156	197c	75b	149b
HD 2967	433	333a	199	378a	217a	189a
LSD_G_ (p=0.05)	n.s	98	n.s.	54	118	40
LSD_T×G_ (p=0.05)	115	n.s.	n.s.	n.s.	44	n.s.

	Pakistan			
	Faisalabad		Gujranwala	Sheikhupura
	2015–2016	2016–2017	2016–2017	2016–2017
Soil/foliar				
LC	2 b	47 c	7 c	28 b
LC +F-CT	*	436 b	253 b	272 a
LSD_T_ (p=0.05)	163 a	521 a	317 a	289 a
Genotype				
Zincol-2016	18	60	51	28
NR-488	44 b	374 a	178	173 b
FSD-2008	97 a	336 ab	203	199 ab
LSD_G_ (p=0.05)	107 a	295 b	197	218 a
LSD_T×G_ (p=0.05)	22	53	n.s.	28

Different letters across a column indicate significant differences according to LSD test (p<0.05). Where the letters are missing, ANOVA revealed the absence of an overall effect. n.s. stands for not significant. *Treatment error0.

LC, local treatment; S-Zn, soil Zn application; F-Zn, foliar Zn application; F-Zn+I, foliar Zn and I (iodine) application; and F-CT, combined foliar treatment of Zn+Se+I.

In India, the highest grain I concentration was recorded in local check genotype HD 2967 which was significantly higher than the HarvestPlus-biofortified genotypes PBW-1-Zn and HPBW 10 in Gurdaspur during 2015–2016 and at all locations during 2016–2017. HarvestPlus wheat genotype NR-488 and local check variety Faisalabad-2008 had similar grain I concentrations across the locations. At Faisalabad during 2015–2016, the genotype NR-488 and local check cv. Faisalabad-2008 contained substantially higher I concentrations in grains than Zincol-2016.

Overall, among the treatments, the highest grain I concentration across all field locations in both countries was found with the F-CT treatment (**Table 10**). All experimental treatments markedly increased the grain I concentration, compared with the local control, at all field locations; however, the magnitude of increase was cultivar- and location-specific (**Table 9**). Among the genotypes studied, the highest I concentration was recorded with F-CT treatment in the genotypes PBW-1- Zn in India and Zincol-2016 in Pakistan, which was significantly higher than I concentration in HPBW 10 (India) and NR-488 (Pakistan), respectively.

**Table 10:** Pooled means of wheat grain I concentration across locations and years in India and in Pakistan in 2016–2017.

Genotype	LC	F-Zn+I	F-CT	Mean
	I Concentration (µg kg^−1^) India			
PBW 1 Zn	18d	290c	385b	231A
HPBW 10	8d	231c	335bc	191B
HD 2967	12d	347bc	516a	292A
Mean	13C	289B	412A	
	Pakistan			
Zincol-2016	28 e	290 d	408 a	242
NR-488	21 e	351 bc	365 b	245
FSD-2008	34 e	320 cd	356 bc	237
Mean	27 C	320 B	376 A	
Country mean	20	305	394	
LSD (p=0.05)	India		Pakistan	
Treatments	128		24	
Genotypes	51		n.s.	
Interaction (T×G)	80		42	

Table legend: LC, local treatment; S-Zn, soil Zn application; F-Zn, foliar Zn application; F-Zn+I, foliar Zn and I (iodine) application; and F-CT, combined foliar treatment of Zn+Se+I

Different letters within a column and across mean values of a country indicate significant differences according to LSD test (p<0.05). Where the letters are missing, ANOVA revealed the absence of an overall effect. n.s. stands for not significant.

### 3.5 Grain selenium

Grain Se concentrations of wheat genotypes increased significantly with the F-CT treatment at all field locations both in India and in Pakistan (**Table 11**). During 2015–2016, wheat genotypes did not differ significantly in grain Se concentration in India. However, during 2016–2017, genotype PBW-1-Zn and HPBW 10 had significantly higher grain Se concentrations than the local check variety HD 2967. During 2015–2016 at Faisalabad, the local check cv. Faisalabad-2008 exhibited the highest Se whereas during 2016–2017, the genotypes differed non-significantly at this location. At Gujranwala, genotypes Zincol-2016 and NR-488 had higher grain Se concentrations than the local check cv. Faisalabad-2008, whereas NR-488 had the highest grain Se concentration among the genotypes. On average, the F-CT increased Se concentration in wheat grains by 106% in India and 123% in Pakistan (**Table 12**). Genotype response to Se micronutrient was similar in India, but F-CT treatment increased the Se concentration in grains of genotype NR-488 higher than the rest of the genotypes in Pakistan. Overall, F-CT showed 112.8% higher grain Se than local control treatment across India and Pakistan.

**Table 11:** Effect of micronutrient treatments on grain Se concentrations of various wheat genotypes grown at different locations of India and Pakistan.

Treatment/genotype	Se concentration (µg kg^−1^) India				
	2015–2016			2016–2017	
	Ludhiana	Gurdaspur	Bathinda	Ludhiana	Gurdaspur
Soil/foliar					
LC	334b	31b	56b	633b	38b
LC +F-CT	578a	381a	213a	871a	214a
LSD_T_ (p=0.05)	52	48	52	126	18
Genotype					
PBW 1 Zn	425	199	197	807a	143a
HPBW 10	506	216	109	749a	133ab
HD 2967	438	205	97	701b	103b
LSD_G_ (p=0.05)	n.s.	n.s.	n.s.	95	32
LSD_(T×G)_ (p=0.05)	n.s.	43	n.s.	n.s.	n.s.

	Pakistan			
	Faisalabad		Gujranwala	Sheikhupura
	2015–2016	2016–2017	2016–2017	2016–2017
Soil/foliar				
LC +LC	254 b	242 b	96 b	86 b
F-CT	460a	396 a	236 a	309 a
LSD_T_ (p=0.05)	28	25	20	45
Genotype				
Zincol-2016	306 c	307	175 a	179 b
NR-488	364 b	340	196 a	262 a
FSD-2008	401 a	310	127 b	151 b
LSD_G_ (p=0.05)	34	n.s.	24	55
LSD_(T×G)_ (p=0.05)	48	n.s.	34	78

Different letters across a column indicate significant differences according to LSD test (p<0.05). Where the letters are missing, ANOVA revealed the absence of an overall effect. n.s. stands for not significant.

S-Zn, soil Zn application; F-Zn, foliar Zn application; F-Zn+I, foliar Zn and I (iodine) application; and F-CT, combined foliar treatment of Zn+Se+I

**Table 12:** Pooled means of wheat grain Se concentration across locations and years in India and Pakistan in 2016-17.

Genotype	LC	F-CT		Mean
	Se concentration (µg kg^−1^) India			
PBW 1 Zn	221	487		354
HPBW 10	214	471		342
HD 2967	220	397		309
Mean	219B	452A		
Pakistan				
Zincol-2016	145 c	296 b		221 b
NR-488	152 c	380 a		266 a
FSD-2008^b^	127 c	265 b		196 c
Mean	141 b	314 a		
Country Mean	180 B	383 A		
LSD (p=0.05)	India		Pakistan	
Treatments	108		25	
Genotypes	n.s.		20	
Interaction (T×G)	n.s.		35	

Different letters within a column and across mean values of a country indicate significant differences according to LSD test (p<0.05). Where the letters are missing, ANOVA revealed the absence of an overall effect. n.s. stands for not significant.

## 4 Discussion

### 4.1 Grain yield

Micronutrient malnutrition in developing countries can be alleviated by cultivation of micronutrient-biofortified staple foods. In the present study, 2.6% higher grain yield in India with soil-applied Zn than with local control treatment (**Table 3**) is attributed to a positive impact of Zn fertilization on the wheat crop when soils are marginally supplied with Zn, as shown before (Ram et al., 2015; Cakmak, 2009; Zou et al., 2012). According to several earlier studies, wheat grain yield can also be increased by foliar application of Zn as well, but most commonly only in soils having very low plant available Zn, such as soils having DTPA-Zn ≤ 0.2 mg kg^−1^ (Graham et al., 1992; Cakmak et al., 1999; Cakmak, 2009; Ram et al., 2015). A slight reduction in wheat grain yield with F-Zn+I or F-CT treatments, across different field locations, might have occurred, possibly due to I toxicity to wheat. Although I is not an essential nutrient for crop plants, it might be beneficial to plant growth, depending on the soil and environmental conditions (Kiferle et al., 2021; Ma et al., 2023). The use of I in nutrient solution in soilless culture (equivalent to 0.13 mg L^−1^) produced higher biomass of leafy vegetables, such as Chinese cabbage, spinach, and lettuce (Zhu et al., 2003; Dai et al., 2004; Blasco et al., 2013). The source of I for foliar spays is also quite important. A higher sensitivity of plants to high doses of soil-applied KI, compared with soil-applied KIO_3_, was observed in rice (Kato et al., 2013), lettuce (Lawson et al., 2015), and strawberry (Li et al., 2017), and KIO_3_ is considered a better source of I for foliar feeding (Cakmak et al., 2017).

The reason for the lower yield performance of the HarvestPlus-developed wheat genotypes compared with the local check variety observed at Gurdaspur in India during 2015–16 could not be determined. One explanation could be related to a possible lower resistance of the tested HarvestPlus wheat lines to leaf rust in this region. Due to its higher humidity and proximity to the Shiwalik Hills, Gurdaspur, is considered as a hot spot for wheat leaf rust. At Bathinda in India, the similar performance of all wheat genotypes might be due to a lack of leaf rust incidence, because this field location had less relative humidity. In Pakistan, in addition to lower yield potential of the HarvestPlus-developed high-Zn wheat genotypes, compared with local check cv. Faisalabad-2008, the HarvestPlus-developed wheat lines (in particular NR-488) were observed to be susceptible to fungal disease loose smut at all three field locations (data not shown).

### 4.2 Grain zinc

Generally, soil application of Zn (S-Zn treatment) resulted in either no or minimal increases in grain Zn concentrations of the tested genotypes in India and Pakistan. Marked increases in grain Zn concentration were, however, recorded with the foliar application of F-Zn, F-Zn+I, and F-CT treatments across all field locations in both the countries (**Tables 5**, **6**). A higher grain Zn concentration might result from more effective translocation of foliar-applied Zn to grains, than the soil-applied Zn. Similar effects of foliar Zn feeding on wheat and rice grain Zn were also reported in earlier field studies across the world (Zou et al., 2012; Ram et al., 2015; Prom-u-thai et al., 2020; Stangoulis and Knez, 2022). Zinc exhibits at least a moderate phloem mobility in plants, so that sufficient foliar supply of Zn at appropriate crop growth stages could significantly increase the grain Zn concentration in crop plants to the desired levels (Erenoglu et al., 2011; Gupta et al., 2016; Cakmak and Kutman, 2018; Kutman, 2023). In wheat and rice, it has been shown that significant increases in grain Zn by foliar Zn application occur if Zn spray takes place at a later, rather than an earlier, growth stage (i.e., during grain-filling period). Maintaining a high pool of available Zn during the seed-filling period, for example through foliar Zn spray, is, therefore, critical to achieving successful enrichments of grains with Zn (Cakmak and Kutman, 2018)

We did not observe an antagonism for micronutrient accumulation in wheat grains when micronutrients are applied together (i.e., F-Zn+I and F-CT). However, check cv. Faisalabad-2008 and HarvestPlus-developed high Zn cv. Zincol-2016 in Pakistan had statistically similar grain Zn concentrations under foliar Zn treatments (F-Zn, F-Zn+I, and F-CT), which reached higher levels than the target value. This highlights the importance of foliar Zn fertilization and demonstrates that the target value of grain concentration could be achieved in the normal wheat cultivar by foliar Zn fertilization. This is of particular importance in the case of growing wheat in cadmium (Cd)- and lead (Pb)-contaminated soils wherein HarvestPlus-developed cv. Zincol-2016 had been reported to accumulate more Cd/Pb in grains than the check cv. Faisalabad-2008 (Hussain et al., 2019). However, the experiment conducted by Hussain et al. (2019) was a pot experiment in which an extremely high Cd application rate was used, raising Cd levels well beyond those typically found in any cultivated soil. When both genotypes were grown in a normal field soil, both genotypes had low and comparable Cd concentrations.

In India, the grain Zn concentration of HarvestPlus-genotype PBW-1-Zn was 6.4 mg kg^−1^ higher than the local check cv. HD 2967. In Pakistan, grains of all HarvestPlus-developed genotypes contained higher grain Zn than local check cv. Faisalabad-2008. This was likely due to the inherent capacity of the HarvestPlus wheat genotypes to accumulate more Zn in grains than the local Pakistan check genotype. Govindan et al. (2012) reported that a number of genotypes developed under HarvestPlus breeding programs are available with required target of Zn concentrations over 40 mg kg^−1^, indicating that it is possible to develop Zn-biofortified varieties with better agronomic traits and grain yield. They also found a positive correlation between Zn and Fe concentration in wheat grains, which suggested a good scope for simultaneous enrichment of both the micronutrients in this staple cereal.

Khokhar et al. (2018) observed that inherent wheat grain Zn concentration variability in different genotypes was between 24.9 mg kg^−1^ to 34.8 mg kg^−1^. The variability in Zn concentration of wheat grains observed in our studies is consistent with the earlier observations of Zhao et al. (2009) who reported that the grain Zn concentration in 150 bread wheat genotypes varied from 14 mg kg^−1^ to 35 mg kg^−1^. Similarly, Morgounov et al. (2007) recorded a wide range of grain Zn concentrations (i.e., 20 mg kg^−1^–39 mg kg^−1^) among 66 wheat genotypes selected from Central Asian Breeding Programs, and grown in Kazakhstan, Kyrgyzstan, and Tajikistan. Compared with the respective check cultivars, increases in grain Zn with F-Zn treatment were higher in both HarvestPlus-developed wheat genotypes in India, but only in NR-488 in Pakistan, indicating synergism between the genetic and agronomic biofortification strategies.

HarvestPlus-developed Zn-biofortified wheat cultivars could significantly contribute to enhancing daily Zn intake requirements. For example, in a very recent study, Lowe et al. (2021) reported that based on an average flour consumption of 224 g day^−1^ for 4 weeks, Zincol-2016 provided an additional daily Zn intake of between 3.0 mg and 6.0 mg for white and whole grain flour, respectively. This resulted in a significant increase, a mean difference of 41.5 μg L^−1^, in plasma Zn concentration. Regular consumption of Zincol-2016 flour increased the daily Zn intake of women of reproductive age by 30%–60%. In Pakistan, the HarvestPlus program also released a new and more promising wheat cultivar Akbar-2019, characterized by much higher grain Zn concentration, high grain yield, and better yield stability (Virk et al., 2021; Wani et al., 2022; and see also www.harvestplus.org/countries/pakistan). It would be interesting to include Akbar-2019 in future agronomic biofortification programs to study its response to soil and foliar application of micronutrients.

### 4.3 Grain iron

Among the applied treatments, the highest grain Fe concentration was recorded with the application of F-CT across all field locations during both years, revealing that foliar cocktail containing Zn, I, and Se fertilizers was effective in enriching wheat grains with Fe as well (**Tables 7**, **8**). Pooled analysis of the data revealed that F-CT treatment led to 19.0% higher grain Fe concentration in India and 16.2% higher Fe concentration in Pakistan, than the respective local control treatments. Even F-Zn and F-Zn+I treatments also resulted in significantly higher grain Fe concentrations. In earlier foliar Zn feeding experiments, Zn application had also shown significant positive effects on grain Fe concentrations in bran and embryo parts of wheat grain, which were not related to any Fe contamination of grain samples through soil dusts or particles (Kutman et al., 2011; Li et al., 2016; Zou et al., 2019). This might have resulted from foliar-applied Zn-induced increase in Fe translocation from shoots to grains. Previously, Kutman et al. (2011) suggested that marked increases in grain Zn associated with foliar Zn spray probably induced production of Zn binding compounds in grain, which possibly act as a sink for Fe translocation and deposition in the grains. It is known that the gene GPC-1 affecting grain protein concentration simultaneously improves Zn and Fe concentrations in grain (Distelfeld et al., 2007). Strongly positive correlations between Zn and Fe in cereal grains have been repeatedly reported in the previous studies (Cakmak et al., 2004; Peleg et al., 2008; Gomez-Becerra et al., 2010; Swamy et al., 2016; Stangoulis and Knez, 2022). These findings indicate that there are probably common genetic and physiological factors affecting gran deposition of Zn and Fe in wheat.

### 4.4 Grain iodine

Iodine is an essentially required micronutrient for humans, and the incidence of widespread I deficiency disorders (IDDs) has been closely studied for decades (Hatch-McChesney and Lieberman, 2022). Use of iodized common salt is an effective strategy to combat I malnutrition. However, many social and scientific factors, such as reduced use of salt because of its adverse effects on health, lack of monitoring of I content in the salts, and stability of I in the iodized salts, have led to a failure of this strategy in effectively mitigating I deficiency in human populations (Diosady et al., 1998; Hatch-McChesney and Lieberman, 2022; Nista et al., 2022). In this scenario, the provision of I-enriched staple cereals, such as wheat, appears to be a more effective and sustainable strategy to address I malnutrition.

In the present study, foliar I-application led to substantial increases in grain I concentration (**Tables 9**, **10**). In Pakistan, F-CT treatment led to a 1,420-fold increase in grain I concentration. HarvestPlus-developed genotype PBW-1-Zn and cv. HD 2967 in India and genotype NR-488 and cv. Faisalabad-2008 in Pakistan accumulated significantly higher grain I concentrations compared with the rest of the genotypes in both the countries. Similar marked increases in the I concentrations of food crops through application of I fertilizers were also shown in rice (Prom-u-thai et al., 2020; Naeem et al., 2022), maize (Xue et al., 2023), and fruits of tomato (Landini et al., 2011; Kiferle et al., 2013) and pepper (Li et al., 2017). Very likely, these substantial enrichments were the consequence of effective translocation of foliar-applied I from wheat leaves to the grains. However, it could also be partially related to a direct contamination of florets and seeds through late season I sprays (Cakmak et al., 2017). In lettuce plants, foliar-sprayed I was effectively translocated to plant roots (Smoleń et al., 2014).

### 4.5 Grain selenium

Selenium is a further critical micronutrient that is essential for humans (Rayman, 2012; Lyons et al., 2005; Jones et al., 2017). There are also some published data indicating importance of Se for crop plants (Ma et al., 2023). In our studies, F-CT treatment enhanced the Se concentration in wheat grains by almost twofold (**Tables 11**, **12**). Thus, similar to Zn enrichment of wheat grains, foliar-applied Se enhanced its accumulation in wheat grains. Slight variations in Se accumulation were observed in different wheat genotypes, but, overall, Se enrichment was almost uniform among the tested wheat genotypes. Similar significant increases in grain Se concentrations have been shown in various staple food crops (Curtin et al., 2006; Chilimba et al., 2012; Mao et al., 2014; Prom-u-thai et al., 2020).

It is interesting to note that in contrast to Zn and Fe (Cakmak et al., 2010), Se is more or less evenly distributed within the grain fractions (Lyons et al., 2005). Zinc and Fe are predominantly localized in the aleurone and embryo parts of wheat grains (Cakmak et al., 2010). However, Rodrigo et al. (2014) showed that only 15% of total grain Se was found in the bran (including seed coat and aleurone) and germ whereas the remaining part of Se was found in white flour, which is the most widely consumed grain part. Also, Lyons et al. (2005) showed that up to 95% of the total Se in wheat grain is present in white flour, meaning that those consuming primarily white flour can also benefit from Se biofortification of wheat grain.

Mao et al. (2014) observed similar enrichments of food grains with Se, Zn, and I when these micronutrients were applied singly or simultaneously as a cocktail. Thus, they advocated that agronomic biofortification of various food crops with Zn, Se, and I can be an effective approach to enrich their edible parts with these micronutrients; however, the effectiveness of biofortification varies with crop species, fertilizer form, and application method of micronutrients.

## 5 Conclusion

This study revealed that foliar applications of Zn, I, and Se at earing and early milk development growth stages are effective in enriching wheat grains with these micronutrients as well as with Fe. Agronomically biofortified wheat grains containing high concentrations of these micronutrients could contribute to the fight against a host of health problems in populations suffering with micronutrient malnutrition. Cereal-based foods made from biofortified grains obtained through the application of micronutrient fertilizers will contain nutritionally significant amounts of micronutrients with high levels of bioaccessibility, demonstrating an effective transfer of Zn, Se, and I from the field to the grain and, ultimately, to the end product. The biofortified wheat genotypes in India were able to compete for grain yield with local check variety HD 2967; however, Zincol-2016 and NR-488 gave lower yields than local check variety Faisalabad-2008. However, in Pakistan, the newly released Akbar-2019 appears to be a much more promising Zn-biofortified wheat with higher Zn and higher yield than well-known local wheat varieties (Ahmad et al., 2019). As shown with high-Zn genotypes PBW-1-Zn in India and NR-488 in Pakistan, foliar Zn spray to Zn-biofortified genotypes provides additional increments in grain Zn, of more than 15 mg per kg grain. Thus, combining agronomic and genetic strategies will raise grain Zn concentrations to over 50 mg kg^−1^. This demonstrates the potential of combining plant genetic- and fertilizer-based approaches to create additive and synergistic impacts for the accumulation of wheat grain Zn, at desirable amounts for human nutrition.

## Data Availability

The raw data supporting the conclusions of this article will be made available by the authors, without undue reservation.
